# Teleportation goes to Hertz rate

**DOI:** 10.1038/s41377-023-01216-0

**Published:** 2023-07-05

**Authors:** Zhihui Yan, Xiaojun Jia

**Affiliations:** State Key Laboratory of Quantum Optics and Quantum Optics Devices, Institute of Opto-Electronics, Collaborative Innovation Center of Extreme Optics, Shanxi University, 030006 Taiyuan, China

**Keywords:** Quantum optics, Quantum optics

## Abstract

Quantum teleportation has been developed to simultaneously realize the Hertz rate and the 64-km distance through fiber channels, which is essential to real-world application of quantum network.

Quantum teleportation is one of the most important protocols in quantum information science, and enables the transfer of an unknown quantum state over long distances by using quantum entanglement resource^1–3^. Thanks to its recent fast development, teleportation-based quantum information science has become a promising field that inspires many important applications. Quantum teleportation enables the remote transfer of quantum state in quantum communication network^4–6^ and the long-range interaction among quantum states in distributed quantum computation^7^. So far, great efforts have been made in quantum teleportation with a variety of quantum systems. Quantum optics-based teleportation offers a promising avenue towards quantum networks, where the quantum states are key resources of quantum information science, and not only coherent states^8,9^ but also nonclassical states^10^ have been experimentally teleported. For practical application, the high-rate quantum teleportation is demanded for effectively transferring quantum state^11,12^. Meanwhile, it is also required to teleport quantum state over remoter users. The transfer distances have been extended over 1400 km with a low-Earth orbit satellite^13^, and over 100 km through commercial optical-fiber networks^14^, respectively. Therefore, it is required to simultaneously realize quantum teleportation with the both long distance and high rate in real-world scenario.

For practical quantum teleportation network, in a newly published paper in *Light: Science & Applications*, the team led by Qiang Zhou from the Institute of Fundamental and Frontier Sciences, University of Electronic Science and Technology of China has reported an experimental realization of a Hertz-rate quantum teleportation system through a real-world fiber network^15^. The techniques of high-performance time-bin entangled source with a periodically poled lithium niobate (PPLN) waveguide and a fully running feedback system for quantum states distribution are employed, thus a weak coherent single photon with decoy state is transferred at a rate of 7.1 ± 0.4 Hz among different real-world buildings connected by 64-km-long fiber channel, as illustrated in Fig. 1. Furthermore, the average single-photon fidelity of ≥90.6 ± 2.6% is experimentally achieved.

**Fig. 1:**
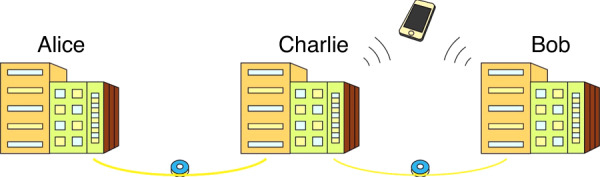
Schematic view of the Hertz-rate metropolitan quantum teleportation. All these three buildings are connected by fibers to construct quantum channels. Alice prepares a weak coherent single-photon state and sends it to Charlie through one quantum channel. Bob generates a pair of entangled signal and idler photons and sends the idler photon to Charlie via another quantum channel. Charlie implements a joint Bell-state measurement and sent this result to Bob via a classical channel. Bob reconstructs the initial state at Alice by a unitary transformation on the entangled signal photon

It is foreseeable that the quantum teleportation can give rise to exciting inspirations for both advanced quantum technology and quantum network applications, as illustrated in Fig. 2 of the quantum teleportation sceneries. The high fidelity, high capacity and quantum memory are demanded in quantum teleportation, besides high rate and long distance as discussed in this work. Quantum network consists of quantum channels and quantum nodes^16,17^. On quantum channel, there are still improvement spaces for high performance quantum teleportation. The rate of quantum teleportation can be increased by improving efficiency and repetition rates of generation, manipulation and measurement. Besides improving indistinguishability, the high-quality quantum light source^18,19^ and entanglement enhancement^20,21^ provide possibility of high-fidelity quantum teleportation. Continuous variable (CV) quantum information processing system benefits from high efficiency generation and detection, as well as unambiguous state discrimination, although its fidelity is limit due to the losses. Meanwhile, discrete variable (DV) system can perform high fidelity quantum information processing as a result of resisting the losses, although it is restricted by probabilistic operations. Thus, the hybrid architecture of both CV and DV approaches may have potential advantages on combination of two approaches^11^. By combining the complex quantum states, such as multiple degrees of freedom and high-dimensional quantum states, quantum teleportation can increase its capacity^22,23^. Furthermore, the distance of teleportation can be improved by integration techniques of free space and fiber channel, and even quantum repeater^4,5^. On the quantum node, various platforms, including atomic ensembles^24^, single atoms^25^, trapped ions^26,27^, solid-state quantum systems^28^, and nuclear magnetic resonance^29^, enable quantum teleportation between matter nodes. In the future, the hybrid approach of these above technologies provides possible way to realize a high-performance quantum teleportation network.

**Fig. 2:**
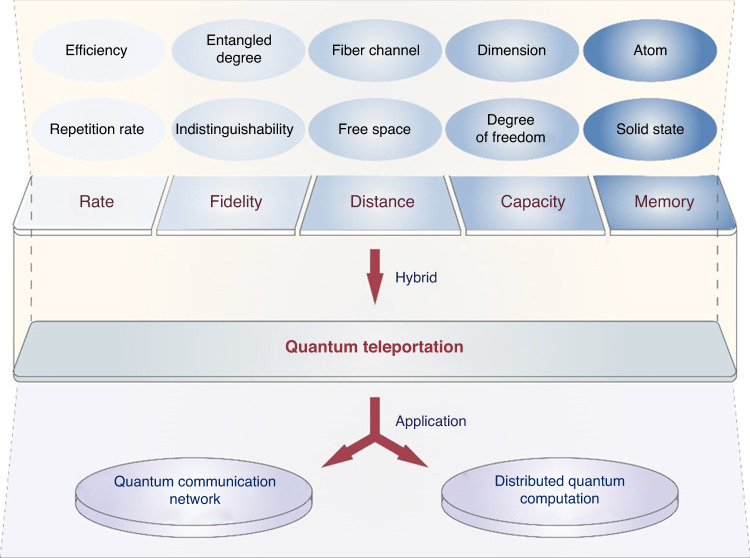
Future vision of the quantum teleportation for quantum network application. The possible approaches for improving the performance of quantum teleportation is on the top part, and the potential applications of quantum teleportation is on the bottom part

Looking forward, while the quantum teleportation establishes an important foundation of quantum network, it also fosters inspirations to future possible applications. Quantum teleportation will play an essential role to realize quantum communication towards global scale^17,18^. Besides, quantum teleportation is potentially applied to distributed quantum computation^7^. Quantum teleportation can distribute local gate operations between distant users, and be used to link the distributed quantum computing units. This work establishes an important step from proof-of-principle demonstrations to real-world applications of quantum teleportation.
